# The polyphenol quercetin induces cell death in leukemia by targeting epigenetic regulators of pro-apoptotic genes

**DOI:** 10.1186/s13148-018-0563-3

**Published:** 2018-11-08

**Authors:** Marisa Claudia Alvarez, Victor Maso, Cristiane Okuda Torello, Karla Priscilla Ferro, Sara Teresinha Olalla Saad

**Affiliations:** Hematology and Transfusion Medicine Center-University of Campinas/Hemocentro-UNICAMP, Instituto Nacional de Ciencia e Tecnologia do Sangue, Rua Carlos Chagas, 480, CEP, Campinas, SP 13083-878 Brazil

**Keywords:** Quercetin, HDACs, DNMTs, Epigenetics, Leukemia

## Abstract

**Background:**

In the present study, we investigated the molecular mechanisms underlying the pro-apoptotic effects of quercetin (Qu) by evaluating the effect of Qu treatment on DNA methylation and posttranslational histone modifications of genes related to the apoptosis pathway. This study was performed in vivo in two human xenograft acute myeloid leukemia (AML) models and in vitro using HL60 and U937 cell lines.

**Results:**

Qu treatment almost eliminates DNMT1 and DNMT3a expression, and this regulation was in part STAT-3 dependent. The treatment also downregulated class I HDACs. Furthermore, treatment of the cell lines with the proteasome inhibitor, MG132, together with Qu prevented degradation of class I HDACs compared to cells treated with Qu alone, indicating increased proteasome degradation of class I HDACS by Qu. Qu induced demethylation of the pro-apoptotic BCL2L11, *DAPK1* genes, in a dose- and time**-**dependent manner. Moreover, Qu (50 μmol/L) treatment of cell lines for 48 h caused accumulation of acetylated histone 3 and histone 4, resulting in three- to ten fold increases in the promoter region of *DAPK1*, *BCL2L11*, *BAX*, *APAF1*, *BNIP3*, and *BNIP3L*. In addition, Qu treatment significantly increased the mRNA levels of all these genes, when compared to cells treated with vehicle only (control cells) (*p < 0.05).

**Conclusions:**

In summary, our results showed that enhanced apoptosis, induced by Qu, might be caused in part by its DNA demethylating activity, by HDAC inhibition, and by the enrichment of H3ac and H4ac in the promoter regions of genes involved in the apoptosis pathway, leading to their transcription activation.

## Background

The myelodysplastic syndromes (MDSs) are a group of diverse and heterogeneous disorders characterized by clonal proliferation, bone marrow failure, and an increased risk of the development of acute myelogenous leukemia (AML) [1]. Leukemia has traditionally been considered to be the consequence of genetic alterations; however, in recent years, a body of evidence has demonstrated that the neoplastic phenotypes, including leukemia, may also be mediated by epigenetic alterations [2–4]. Epigenetics, broadly, refers to stimuli-triggered changes in gene expression due to processes that arise independentless of changes in the underlying DNA sequence. Some of these processes include DNA methylation [5], histone modifications and chromatin-remodeling proteins [6], and DNA silencing by noncoding RNAs (ncRNA) [7]. The reversability of epigenetics makes this mechanism a potential target for novel therapeutic approaches.

Quercetin (Qu) is an important dietary flavonoid, present in different vegetables, fruits, nuts, tea, red wine, and propolis [8–10]. Its study as potential therapeutic agent is assuming importance considering its involvement in the suppression of many tumor-related processes including oxidative stress, apoptosis, proliferation, and metastasis. Qu has also received attention as a pro-apoptotic flavonoid with a specific and almost exclusive effect on tumor cell lines rather than normal, non-transformed cells [11].

A previous study from our group showed that Qu caused pronounced apoptosis in leukemia cells, in vivo and in vitro (xenograft model), followed by *BCL-2*, *BCL-XL*, *MCL-1* downregulation, *BAX* upregulation, and mitochondrial translocation, triggering cytochrome c release and caspase activation [12]. In the present study, we investigated the molecular mechanisms underlying the pro-apoptotic effects of Qu by evaluating the effect of Qu treatment on DNA methylation and posttranslational histone modifications of genes related to the apoptosis pathway. Qu treatment of the myeloid leukemia cells, in vitro or in a human tumor xenograft, induced apoptosis, in part, through the reversal of epigenetic alterations.

## Results

### Gene-specific promoter methylation of apoptosis-related genes

**Fig. 1:**
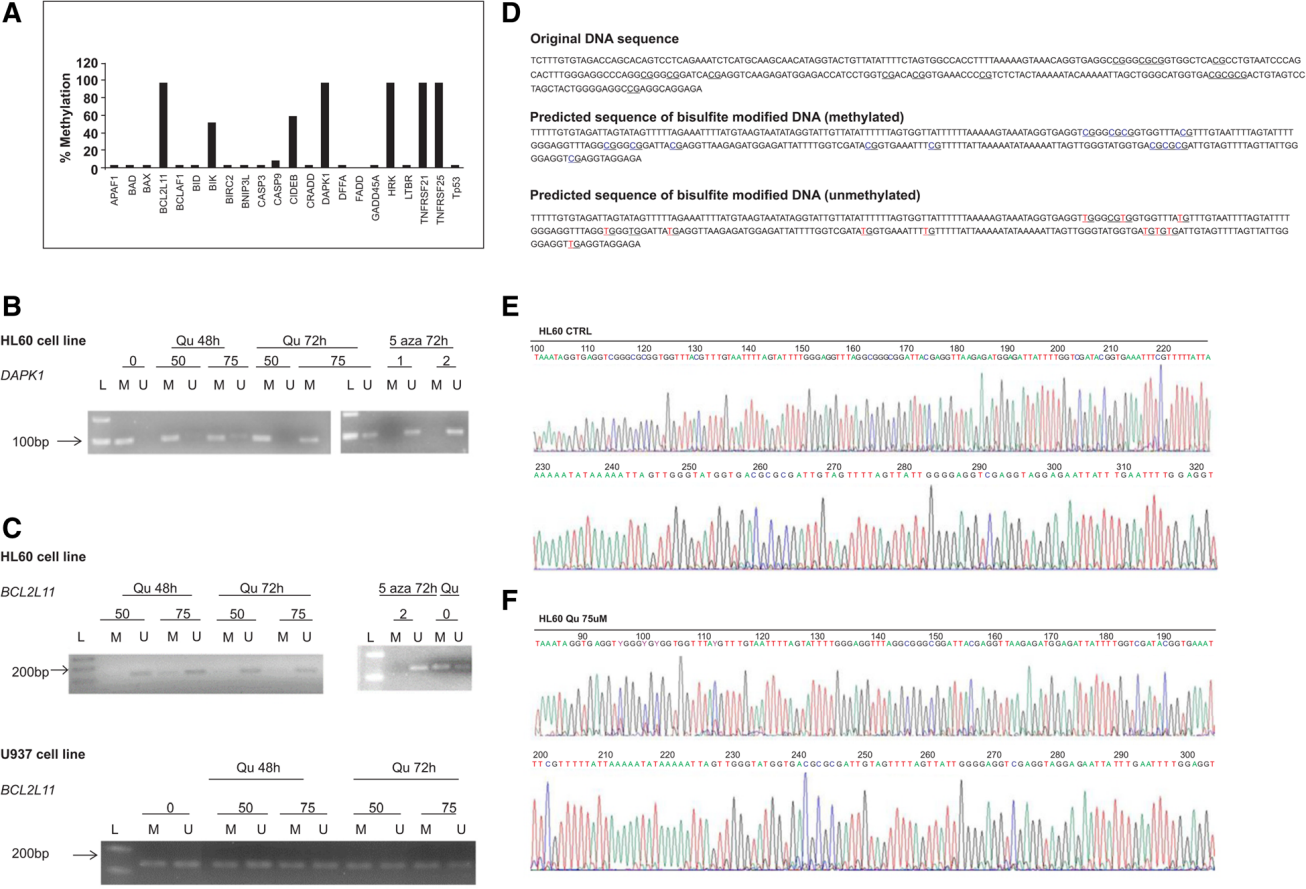
Methylation analysis. **a** Methylation screening of HL60 cell line. **b** *DAPK1* methylation-specific polymerase chain reaction analysis. HL60 cells treated with 50 and 75 μmol/L Qu for 48 and 72 h and 1 and 2 μmol/L 2-deoxy-5-aza cytidine for 72 h. Lane L: ladder; lane M: amplified product with primers for methylated sequences (106 bp); lane U: amplified product with primers for unmethylated sequences (98 bp). **c** *BCL2L11* methylation-specific polymerase chain reaction analysis. HL60 and U937 cells treated with 50 and 75 μmol/L Qu for 48 and 72 h and 2 μmol/L 2-deoxy-5-aza cytidine for 72 h. L: ladder; lane M: amplified product with primers for methylated sequences (139 bp); lane U: amplified product with primers for unmethylated sequences (139 bp). **d** Bisulfite sequencing of *DAPK1* promoter: original DNA sequence, bisulfite-modified DNA sequence (methylated), and bisulfite-modified DNA sequence (unmethylated). **e** Electropherogram for HL60 cell line. **f** Electropherogram for HL60 treated with 75 μmol/L Qu for 72 h. Y represents heterozygote C/T double peaks, indicating partial methylation

We examined the DNA methylation status at the promoter CpG islands of 24 apoptosis-related genes in the HL60 cell line. Of the 24 genes assayed in the cell line, the extent of promoter methylation in five genes (*BCL2L11*, *DAPK1*, *HRK*, *TNFRSF21*, *TNFRSF25*) exceeded 90% (Fig. 1a). In order to determine whether quercetin affected CpG methylation, we further validated the promoter methylation of *BCL2L11* and *DAPK1* by MSP-PCR in samples treated with 50 and 75 μmol/L of Qu for 48 and 72 h. After 72 h of Qu treatment, there was partial demethylation of *BCL2L11* and *DAPK1* gene promoters in HL60 cells (Fig. 1b, c). The partial demethylation of DAPK1 promoter was confirmed by bisulfite sequencing (Fig. 1d-f). Concentrations of 1 and 2 μM concentration of 5-aza-dC were chosen as positive control. The U937 cell line was also treated with Qu (same concentrations and period of time as for HL60 cells). This cell line was unmethylated in the promoter region of *DAPK1* and hemimethylated in the promoter region of *BCL2L11*. No demethylation was observed after Qu treatment (Fig. 1c).

### Quercetin downregulates DNMTs and STAT3

**Fig. 2:**
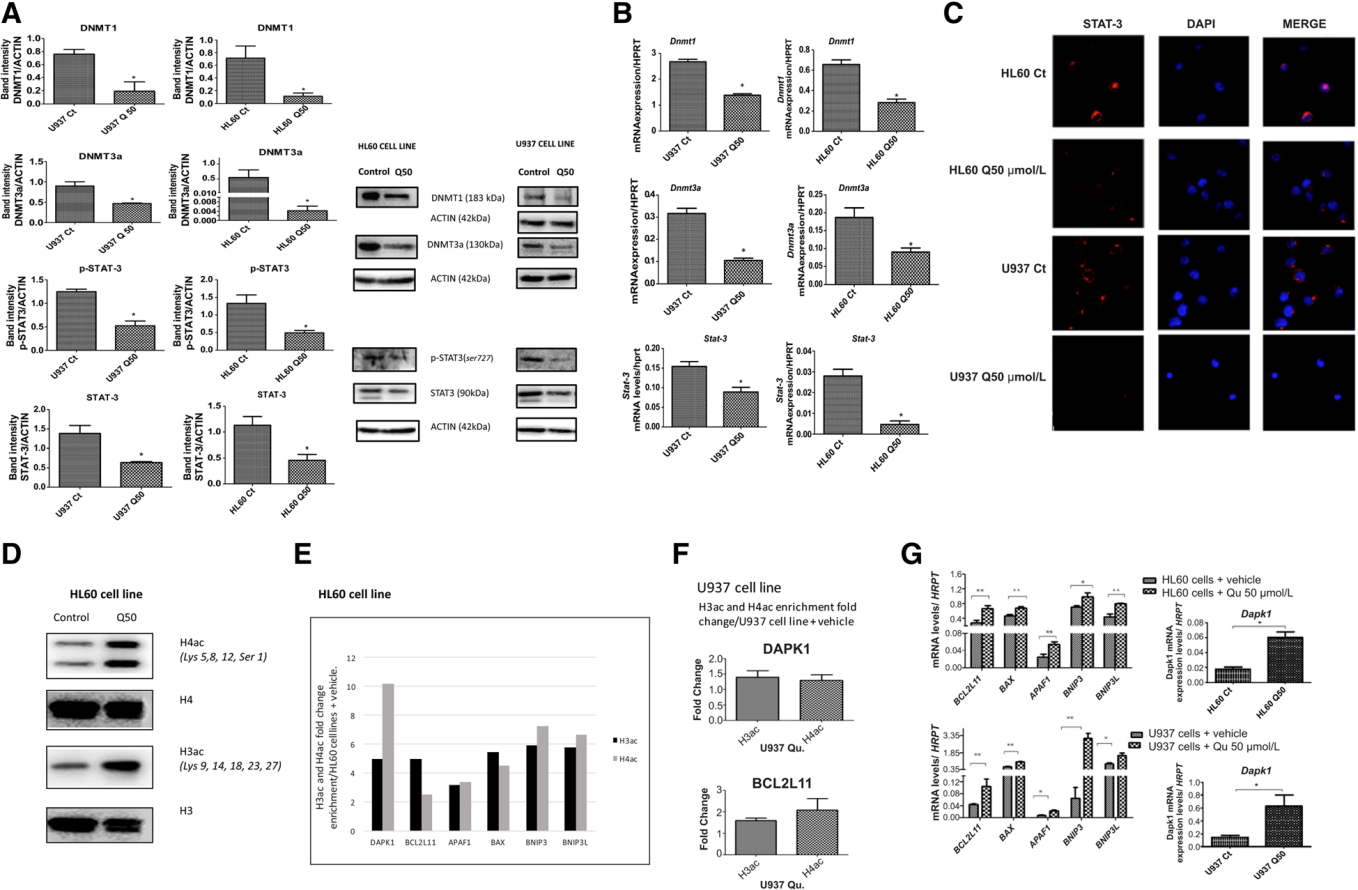
Quercetin treatment decreases DNMTs, in STAT3-dependent manner, increases global acetylation of H3 and H4, and increases the enrichment of acetylated histone H3 and H4 to the promoters of genes related to the apoptosis pathway. **a** HL60 and U937 cell lines were treated with 50 μmol/L Qu for 48 h. Western blotting was performed for DNMT1, DNMT3a, p-STAT3, and STAT3 proteins. Values are means ± SD of three independent assays. *p < 0.05 when compared to cell lines treated with vehicle only. One experiment in three is shown. **b** The mRNA values are expressed as mean ± SD of three independent experiments. *p < 0.05; ** *p* < 0.005 when compared to HL60 or U937 cells treated with vehicle only. **c** Confocal microscopy view of HL60 and U937 cells treated with 50 μmol/L Qu for 48 h, incubated with STAT3 antibody and probed with Alexa Fluor 555 (red)-labeled secondary antibody showing a decreased STAT3 expression after treatment. DAPI was used to stain nuclei. **d** Western blotting analysis of acid extracted proteins of HL60 cell line exposed to 50 μmol/l Qu for 48 h. Qu treatment increases acetylated levels of H3 and H4. One representative experiment in three is shown. **e** Treatment of HL60 cells with 50 μmol/L Qu for 48 h. *Apoptosis ChIP human PCR array* for association of acetylated histone H3 and H4 with the promoters of *DAPK1*, *BCL2L11*, *APAF1*, *BAX*, *BNIP3*, and *BNIP3L* was performed; details are provided in the “Methods” section. **f** Chromatin immunoprecipitation assay was performed for association of acetylated H3 and H4 histones with the promoters of *DAPK1* and *BCL2L11* in U937 cells treated with 50 μmol/L Qu for 48 h. Qu treatment caused increased association of acetylated histones H3 and H4 to the promoters of *DAPK1* and *BCL2L11*. **g** *BCL2L11*, *BAX*, *APAF1*, *BNIP3*, and *BNIP3L* mRNA expression levels of HL60 and U937 cells treated with 50 μmol/L Qu for 48 h. The mRNA values are expressed as mean ± SD of three independent experiments. *p < 0.05, **p < 0.005, when compared to HL60 or U937 cells + vehicle

Since Qu induced partial demethylation in the promoter regions of highly methylated genes, Western blot analyses using anti-DNMT1 (DNA methyltransferase 1) and anti-DNMT3a (DNA methyltransferase 3a) antibodies were performed. Qu treatment decreased the levels of both proteins. Next, since the STAT3 pathway direct regulates DNMTs [13], we investigated whether Qu treatment modulates these proteins. Western blot analysis, RT-PCR, and confocal microscopy showed that Qu treatment downregulated STAT3 expression and phosphorylation (*p < 0.05). These data provide evidence that Qu downregulates DNMTs through STAT3 pathway (Fig. 2a–c).

### Quercetin induces H3 and H4 global acetylation

Like DNA methylation, the acetylation or methylation of histone proteins comprise major epigenetic processes on the chromatin structure, altering nucleosomal architecture and leading to gene activation or repression. In order to determine whether Qu also affects histones, we determined the effect of Qu treatment on global acetylation of histones 3 and 4. For this purpose, Western blotting of the acid extracted proteins of the HL60 cell line were exposed to 50 μmol/L Qu for 48 h and western blotting was performed. After Qu treatment, a global increase in H3ac and H4ac was observed compared to cells treated with vehicle only (Fig. 2d).

### Quercetin enriched H3ac and H4ac in the promoter region of the apoptosis pathway genes and increased their transcription levels

We then wondered whether this increase in acetylation of H3 and H4 also occurred at promoter regions of genes involved in the apoptosis pathway. To verify this, an *Apoptosis ChIP human PCR array* (SAbiosciences, Qiagen) was performed. HL60 cells treated with 50 μmol/L Qu, for 48 h induced a three- to ten fold enrichment of H3ac and H4ac in the promoter regions of *APAF1*, *BAX*, *BCL2L11*, *BNIP3 BNIP3*, and *DAPK1* (Fig. 2e). We further analyzed the mRNA expression levels of these genes in HL60 and U937 samples treated with 50 μmol/L Qu for 48 h. Qu treatment significantly increased the mRNA levels of all these genes in both cell lines, when compared to control cells (**p < 0.005; *p < 0.05) (Fig. 2g). As the *DAPK1* gene promoter is unmethylated and Qu treatment did not induce demethylation of promoter region of *BCL2L11* in the U937 cell line, furthermore, Qu treatment upregulated mRNA expression levels of *DAPK1* and *BCL2L11*, we proceeded to investigate whether Qu treatment also induced an enrichment of H3 and H4 acetylation in the promoter regions of *DAPK1* and *BCL2L11* in U937 cell line.

Using anti acetylated histone H3 and H4 antibodies, followed by RT-PCR with specific primers for *DAPK1* and *BCL2L11* promoters, a chromatin immunoprecipitation assay was performed. As shown in Fig. 2f, Qu treatment resulted in an increase in the amount of acetylated H3 and H4 associated with both *DAPK1* and *BCL2L11* promoters.

**Fig. 3:**
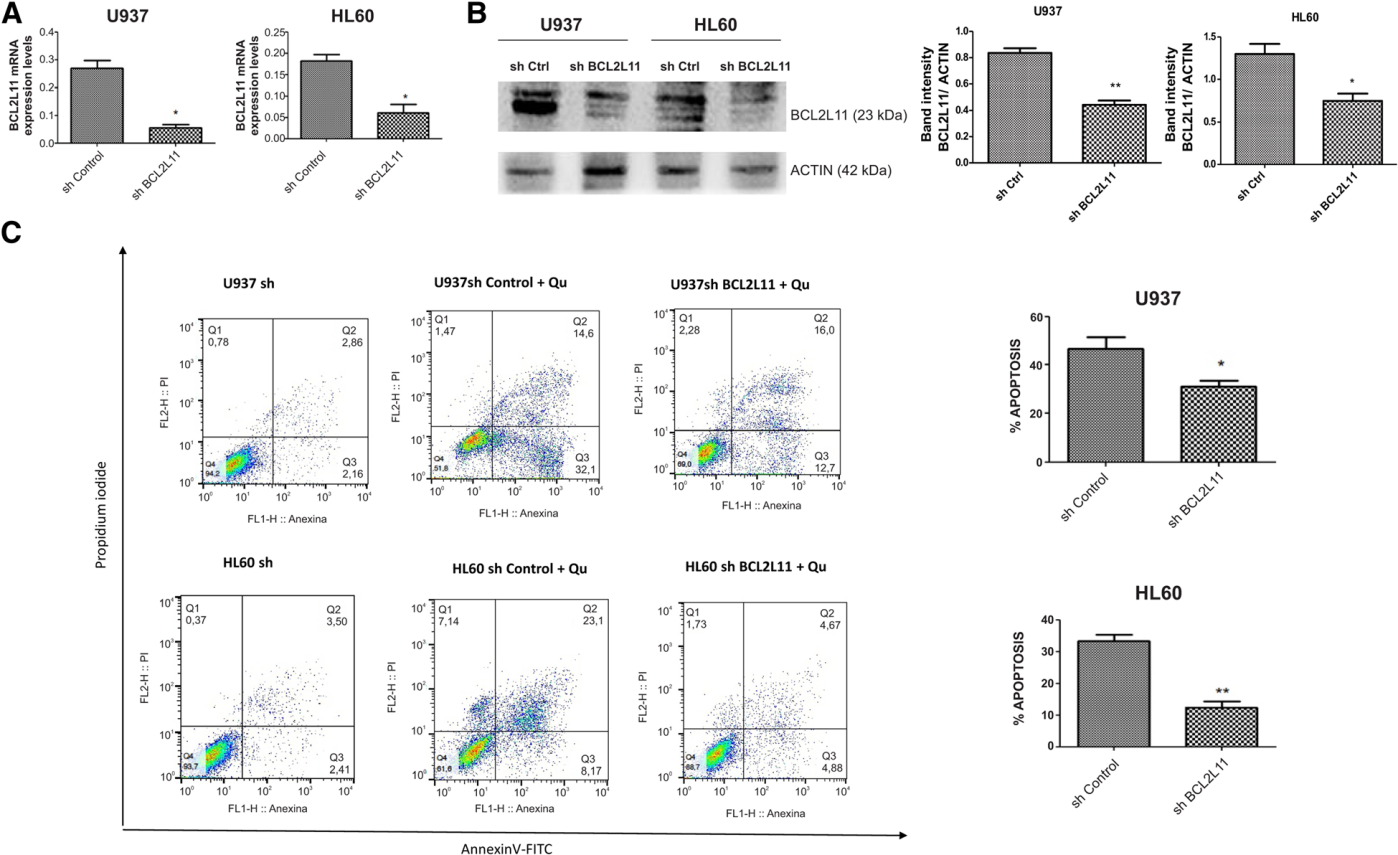
Impact of shBCL2L11 leukemia cell lines on apoptosis induced by quercetin. **a** BCL2L11 mRNA relative expressions in sh BCL2L11 U937 and HL60 cell lines. **b** Western blotting was performed for BCL2L11in sh control and sh BCL2L11 U937 and HL60 cell lines. **c** sh control and sh BCL2L11 U937 and HL60 cell lines were treated with 50 μmol/L Qu for 48 h, after treatment cells were stained with annexin V/PI. The percentage of apoptotic cells were determined by flow cytometry. Values are expressed as mean ± SD. *p < 0.05; **p < 0.005 when compared to sh control cell lines. All data were representative of three independent experiments

To confirm that Qu effect on apoptosis was via *DAPK1* and *BCL2L11*, we proceeded to stably inhibit these genes in the cell lines. Further, the transduced cell lines were treated with 50 μmol/L of Qu for 48 h, and apoptosis was detected by flow cytometry. It was observed that in both *BCL2L11* shRNA-transduced cell lines, Qu induced apoptosis at a lesser extend when compared to control shRNA-transduced cell lines (*p < 0.05; **p < 0.001) (Fig. 3a–c). It was not possible to accomplish *DAPK1* inhibition. For both cell lines, the shRNA lentiviral particle caused too much damage and cells did not recover from transduction.

### Quercetin decreases the protein expression of class I histone deacetylases (HDACs) in leukemia cells

**Fig. 4:**
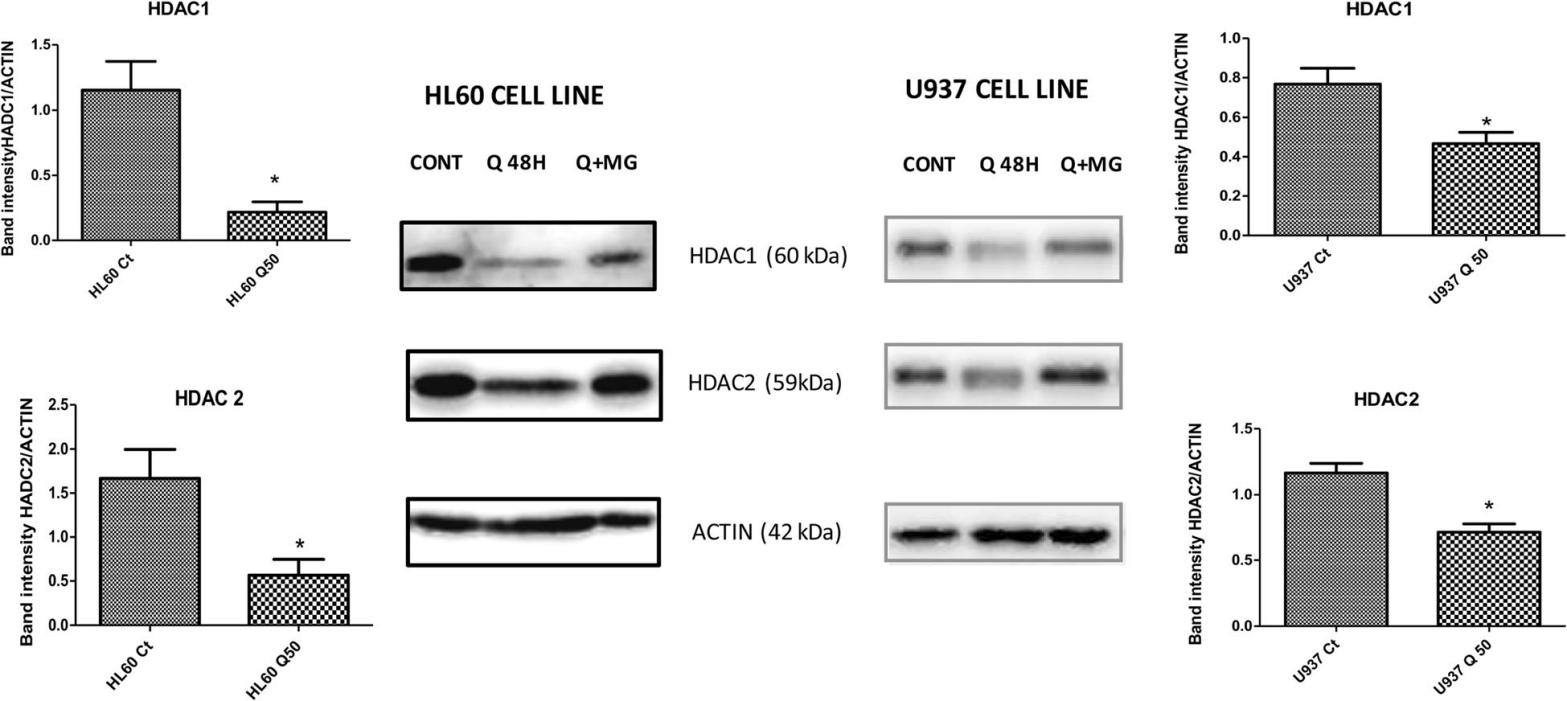
Effect of quercetin treatment on class I HADC level and on the induction of proteasome degradation in leukemia cells. HL60 and U937 cell lines were treated in duplicate with 50 μmol/L Qu for 40 h, and one group was treated later with 10 μmol/L of MG132 (an inhibitor of proteasome) for an additional 8 h. Western blotting was performed for class I HDACs. One representative experiment of three is shown. Values are means ± SD of three independent assays. *p < 0.05

Given the data described herein and the fact that HADCs are found in corepressor complexes, we investigated whether the global increment of the acetylation of H3 and H4 affected the expression levels of HADCs. To establish whether Qu treatment alters the protein expression of class I HADCs, we performed Western blot analysis on total cell lysates of HL60 and U937 cells treated with 50 μmol/L Qu for 48 h. Exposure of cells to Qu caused a decrease in the levels of HADCs 1 and 2 (*p < 0.05) (Fig. 4) but not of HDAC 3 and 8 in both cell lines (data not shown).

### Quercetin caused proteasome-mediated protein degradation of HADCs in leukemia cells

Furthermore, we determined whether proteasome-mediated degradation was involved in the downregulation of HADCs in cells exposed to Qu. HL60 and U937 cell lines were exposed to 50 μmol/L Qu for 40 h in duplicate, followed by the addition of 10 μmol/L MG132 (an inhibitor of proteasome) to one group and incubated for an additional 8 h. Compared to cells treated only with Qu, MG132 caused a significant increase in HADC expression in both cell lines, demonstrating that Qu treatment downregulates these proteins through proteasome degradation (Fig. 4).

### Quercetin downregulates DNMTs and HADCs at the protein levels, in xenograft models

**Fig. 5:**
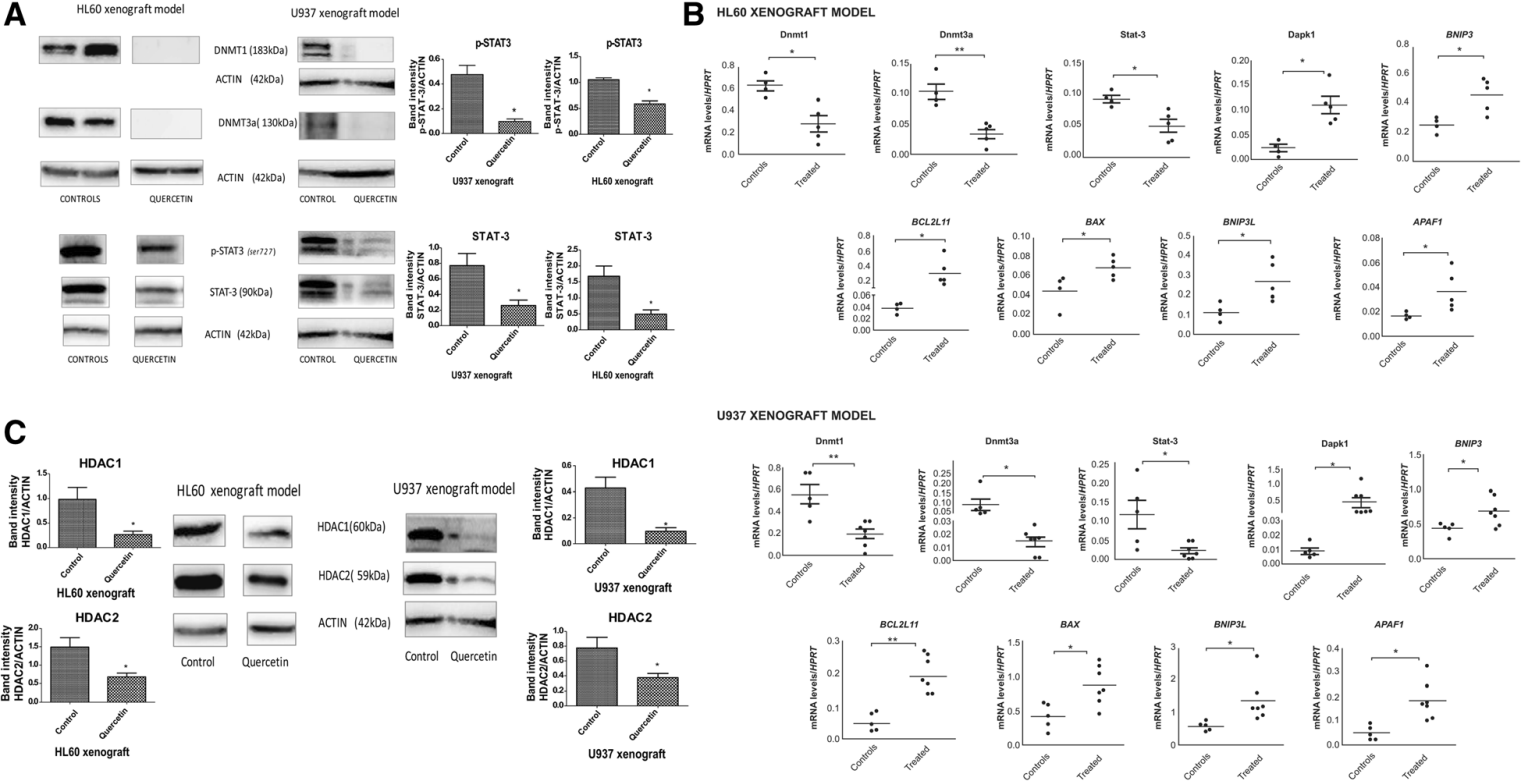
Quercetin treatment decreased DNMTs and class I HDACs protein expression in samples from xenograft models. **a** Xenograft model with HL60 and U937 cell lines. HL60 or U937 cells (1 × 10^7^) were injected s.c. into the flank of NOD/SCID mice. After the tumor grew to about 100 to 200 mm^3^, mice were treated without or with quercetin (120 mg/kg), once every 4 days. The control group received equal amounts of vehicle solution, as indicated in the “Methods” section. After 21 days of treatment, tumors were harvested and Western blotting of DNMT1, DNMT3a, p-STAT3, and STAT3 was performed. Values are expressed as mean ± SD. *p < 0.05;**p < 0.005, when compared to control group treated with vehicle only (HL60 xenograft model: control group *n* = 4; treated group *n* = 5; U937 xenograft model: control group *n* = 5; treated group *n* = 7). **b** Quercetin downregulates *DNMT1*, *DNMT3a*, and *STAT3* and upregulates *DAPK1*, *BCL2L11*, *APAF1*, *BAX*, *BNIP3*, and *BNIP3L* mRNA expression levels. mRNA levels are expressed as mean ± SD.*p < 0.05;**p < 0.001 when compared to control group (HL60 xenograft model: control group *n* = 4; treated group *n* = 5; U937 xenograft model: control group *n* = 5; treated group *n* = 7). **c** After 21 days of treatment, tumors were harvested and Western blotting of HDAC1 and 2 was performed. Values are expressed as mean ± SD. *p < 0.05; **p < 0.005 when compared to control group treated with vehicle only (HL60 xenograft model: control group *n* = 4; treated group *n* = 5; U937 xenograft model: control group *n* = 5; treated group *n* = 7)

Our in vitro results encouraged us to proceed with an in vivo model; mice were subcutaneously engraftment with HL60 and/or U937 cells, as described in the “Methods” section. Accordingly, Qu treatment almost eliminated DNMT protein levels, and this occurred in part through the downregulation of STAT3 and p-STAT3 at the protein and message levels (*p < 0.05;**p < 0.005) (Fig. 5a). mRNA expression levels of *DAPK1*, *BCL2L11*, *BAX*, *BNIP3*, *BNIP3L*, and *APAF1* were significantly upregulated compared to those of the control mice (*p < 0.05; **p < 0.005) (Fig. 5b). Moreover, in treated animals, decreased protein levels of HDAC 1 and 2 were also observed (*p < 0.05; **p < 0.005) (Fig. 5c).

## Discussion

Epigenetic mechanisms involving modifications in the DNA (methylation) and histones (acetylation, methylation, among others) and their corresponding effects on gene regulation have gained considerable interest. The pattern of modifications on histones and DNA constitutes an epigenetic program that is unique for the cell type and is replicated during successive cell divisions. Alterations of these patterns, particularly in the promoter region of genes, can have profound effects on gene expression. Studies from various cancers have revealed that these alterations affect genes involved in different cellular pathways including apoptosis. Cancer cells have the ability to avoid apoptosis, and this is considered to be one of the prime factors that aid the evolution of cancer [14].

Previously, we have shown that Qu upregulated the expression of pro-apoptotic proteins [12]. Then, in this study, we first performed a screening of the promoter methylation status of 24 apoptosis-related genes in human HL60 cells. The analysis revealed that five of these genes were highly methylated, including *BCL2L11*, *DAPK1*, *HRK*, *TNFRSF21*, and *TNFRSF25*. Furthermore, we validated the methylation pattern of *BCL2L11* and *DAPK1* using MSP-PCR in samples treated with Qu and observed that the treatment with Qu totally and partially demethylated *BCL2L11* and *DAPK1*, respectively. This demethylation was both dose and time dependent.

*DAPK1* (death-associated protein kinase 1) is a pro-apoptotic gene that induces cellular apoptosis in response to internal and external apoptotic stimulants [15]. Therefore, silencing of *DAPK1* may result in uncontrolled cell proliferation, indicating that this gene may have a role in tumor suppression. Several studies have demonstrated promoter methylation of *DAPK1* in different types of cancer such as renal [15] and cervical cancer [16], B cell lymphoma [17], myelodysplastic syndrome, acute myeloblastic leukemia [18], and chronic myeloid leukemia [19–21]. Our data show that Qu partially demethylates the promoter region of this gene in both a dose- and time-dependent manner, and this may contribute to the increased apoptosis observed in a previous study realized by our group [12]. Consistent with the demethylating effect of Qu, a previous study reported that different concentrations of Qu partially reverted *P16INK4a* promoter methylation and increased its expression in RKO cells after 5 days of treatment [22].

DNA methylation at the cytosine 5 nucleotide is catalyzed by DNA methyltransferases (DNMTs). This family of proteins is vitally important in epigenetic regulation to modulate gene expression [23]. In our study, Qu treatment almost eliminated DNMT1 and DNMT3a at the protein and message levels, in vitro and in human xenograft models. A previous study, in a gastric cancer cell line, showed that quercetin and isoliquiritigenin decreased DNMT1 and DNMT3a protein levels, causing slight demethylation of the promoter region of the BCL7A gene [24]. We further investigated the expression levels of p-STAT3 and STAT3. STAT3 is a member of a family of transcription factors that regulates proliferation, apoptosis, differentiation, and oncogenesis, and it also regulates DNMT transcription [13]. We found that Qu decreased STAT3 at the protein and at the message level and p-STAT3 at the protein level. The decrease in STAT3 was also confirmed by confocal microscopy. Unlike this previous study [24], we observed that Qu decreased DNMTs in a STAT3-dependent manner, demonstrating that the mode of action of specific natural compounds depends on their cellular origin.

*BCL2L11* (BCL2-interacting mediator, (*BIM*)) is a member of the BH3-only death activator family and a key determinant of cell fate upon cytokine withdrawal. Its expression is regulated by transcriptional and posttranscriptional mechanisms [25]. *BCL2L11* downregulation has a central role in the survival of clonal progenitors of chronic myeloid leukemia (CML), and this low expression was ascribed to DNA hypermethylation at the gene promoter [26–28]. Moreover, *BCL2L11* re-expression has a key role in BCR-ABL1-expressing cell apoptosis in response to imatinib (IM) [29]. We also observed an upregulation of mRNA levels of this gene in vitro and in vivo (xenograft models), induced by Qu treatment. However, in xenograft models, this upregulation was independent of the demethylation of the promoter region of the gene. This upregulation may be the consequence of the augmentation in H3ac and H4ac observed in the promoter region. Moreover, a study in NPM/ALK+ anaplastic large cell lymphoma (ALCL) cell lines and NPM/ALK+ ALCL lymph node biopsies showed that *BCL2L11* is epigenetically silenced and that treatment with the deacetylase inhibitor trichostatin A restores histone acetylation, strongly upregulates *BCL2L11* expression, and induces cell death [30]. In addition, a study realized by Lee et al. [31] showed that Qu induced apoptosis in leukemia HL60 cells by enhancing the expression of Fas-L, in part through the promotion of H3 acetylation.

We observed in vitro that 50 μmol/L Qu increased the enrichment of H3ac and H4ac by three- to ten fold, after 48 h, in the promoter region of *DAPK1*, *BCL2L11*, *BAX*, *BNIP3*, *BNIP3L*, and *APAF1*, compared to control cells. Further, we analyzed mRNA levels of these genes in vitro and in vivo (in samples from xenograft models) and observed that all of them were upregulated, compared to control samples. It is known that histone acetylation mainly occurs at the promoter regions of genes in the process of transcription, whereas histone deacetylation cooperates with gene silencing. Histone deacetylases (HDACs) deacetylate lysine residues of histone tails leading to condensation and closed chromatin formation and transcriptional repression [32].

In the present study, we showed that Qu treatment decreased HDAC1 and HDAC2 protein levels in vitro and in vivo, in the xenograft models, and induced its proteasomal degradation. Many important molecular cell processes are performed by large multisubunit protein complexes, and HDACs do it in the same way. HDAC1 and 2 exist together in at least three distinct multiprotein CoREST complexes [33], and these corepressor complexes are recruited by specific DNA sequences to promoter regions, resulting in transcriptional repression [34].

A potential mechanism to induce apoptosis through histone deacetylase inhibitors (HDACi) is the upregulation of apoptotic genes. HADCi can activate components of the intrinsic pathway, including *BAX* and *APAF1*. APAF1 has been shown to be upregulated by HDACi including suberoylanilide hydroxamic acid SAHA [35, 36]. APAF1 binds to cytochrome C, forming the apoptosome and initiating the caspase cascade. BAX is a cytosolic protein that undergoes conformational change during apoptosis and migrates to the mitochondria, while APAF1 cooperates with the release of cytochrome c. In addition, a recent study showed that two HDACi, kendine 92 and SAHA, induced apoptosis in CLL cells by increasing *BAD*, *BNIP3*, *BNIP3L*, *BIM*, *PUMA*, and *AIF* mRNA expression levels and decreasing expression of *BCL-W*, *BCL-2*, *BFL-1*, *XIAP*, and *FLIP*. Thus, this study indicates global changes in the apoptosis mRNA expression profile, consistent with the apoptotic outcome [37]. Moreover, Qu has also been shown to inhibit cell cycle and induce apoptosis through inhibition of HDAC and DNMT1, thus suppressing tumor growth and angiogenesis in an induced model of hamster buccal pouch carcinoma [38].

## Conclusions

In summary, our results showed that enhanced apoptosis, induced by Qu, might be caused in part by its DNA demethylating activity, by HDAC inhibition, and by the enrichment of H3ac and H4ac in the promoter regions of genes involved in the apoptosis pathway, leading to their transcription activation. Our results provide the first evidence that Qu acts as an inhibitor of the expression of class I HDAC in leukemia cells. We have also demonstrated that this inhibitory effect of class I HDACs by Qu is due to increased proteasomal degradation. The decrease in HDAC expression coincides with increased global as well as local acetylation of H3 and H4 on the *DAPK1* and *BCL2L11* promoters in both cell lines. Further studies on the effect of Qu on histone acetyltransferases (HATs) and HDAC activity as well as other affected molecular pathways in leukemia cells are encouraged.

## Methods

### Reagents and antibodies

Quercetin (> 98% pure) was obtained from Sigma Chemical Co. Antibodies used were as follows: DNMT1, STAT3, HDAC1, HDAC2, acetylated H4 (*Ser 1*/*Lys5*/*Lys 8*/*Lys12*) actin, DAPK1, BCL2L11, and control shRNA (h) lentiviral particles from Santa Cruz Biotechnology. DNMT3a and p-STAT3 were from Cell Signalling Technology. Acetylated H3 (*Lys 9*/*Lys 18*/*Lys 23*/*Lys 27*), H3, and H4 were from Abcam Inc. Anti-rabbit, anti-mouse, and anti-goat per-oxidase-conjugated antibodies were from KPL, Inc. DAPI, Alexa Fluor 555, and Alexa Fluor 488 molecular probes were from Invitrogen. The FITC–Annexin V Apoptosis Detection Kit I was from BD Pharmingen.

### Cell lines and treatment

HL60 and U937 cell lines were purchased from the American Type Culture Collection (ATCC, Philadelphia, PA) and were cytogenetically tested and authenticated before being frozen. HL60 and U937 cells were cultured in RPMI-1640 medium containing 10%n FBS, in a 37 °C humidified atmosphere containing CO_2_. Qu (> 98% pure, Sigma Chemical Co) was dissolved in (DMSO) at a final concentration of 0.1% (*v*/*v*) in RPMI. The cells were treated with Qu at 50 and 75 μmol/L for 48 or 72 h. Control cells were treated with vehicle alone.

### shRNA lentivirus particle transduction

HL60 and U937 cells were infected with DAPK1 or BCL2L11 or control shRNA lentivirus particles in the presence of 5 μg/mL Polybrene (Sigma). Infected cells were selected for 14 days in the presence of 2 μg/mL puromycin (Sigma). Expression of DAPK1 and BCL2L11 in infected cells were verified by real-time PCR (RT-PCR) and western blot analysis.

### Detection of apoptosis by flow cytometry

HL60 and U937 cells transduced with shRNA-targeted *BCL2L11* were seeded on 12-well plates and treated with 50 μmol/L Qu. After 48 h, the cells were washed twice with ice-cold PBS and resuspended in a binding buffer containing 1 mg/mL propidium iodide (PI) and 1 mg/mL FITC-labeled annexin V. All specimens were analyzed on FACSCalibur after incubation for 15 min at room temperature in a light-protected area. Ten thousand events were acquired for each sample.

### Confocal microscopy

Control and treated cells were fixed with 4% PFA (paraformaldehyde) at room temperature for 15 min. Fixed cells were permeabilized with perm buffer (0.2% saponin and 0.1% BSA), blocked with 5% BSA, and incubated overnight in anti-Stat3 antibody at 4 °C. Cells were washed and incubated again with Alexa Fluor 455-conjugated secondary antibody for 2 h at room temperature along with nuclear stain DAPI (4′,6-diamidino-2-phenylindole). Confocal imaging was performed with a Zeiss LSM 710 NLO laser scan confocal microscope using a × 63 objective magnification.

### Methylation analyses

#### Epitec Methyl II PCR Array Human Apoptosis

For the initial screening of the methylation profile of HL60 cell line, the *Epitec Methyl II PCR Array Human Apoptosis* was used. Briefly, input genomic DNA was aliquoted into four equal portions and subjected to mock (Mo), methylation sensitive (Ms), methylation dependent (Md), and double (Msd) restriction endonuclease digestion. After digestion, the enzyme reactions were mixed directly with qPCR master mix and aliquoted into a PCR array plate containing pre-dispensed primer mixes. The plate was run then in real-time PCR using specified cycling conditions. The relative fractions of methylated and unmethylated DNA were subsequently determined by comparing the amount in each digest with that of a mock (no enzymes added) digest using the ΔCt method.

#### Bisulfite treatment and methylation-specific PCR (MSP-PCR)

Bisulfite treatment was performed on 1 μg of DNA previously extracted from cells treated with Qu and from frozen samples from xenograft models using the EpiTect Bisulfite kit (Qiagen, Valencia, CA, USA). Methylation-specific PCR (MSP) [39] was performed for the promoter region of *DAPK* and *BCL2L11* with primers that were specific for the methylated or unmethylated sequences (M or U sets, respectively; Table 1). The reaction products were separated by electrophoresis on 2% agarose gels.

**Table 1:** Primers used for MSP-PCR and real-time PCR

Genes		Primer sense (5′–3′)	Primer antisense (5′–3′)
*BCL2L11*	M	AGTATTTTCGGTAAATAATGGGGT	GAATAAATCAAAAACTCCCAACG
	U	GTATTTTTGGTAAATAATGGGGTTG	CAAATAAATCAAAAACTCCCAACA
*DAPK1*	M	GGATAGTCGGATCGAGTTAACGTC	CCCTCCCAAACGCCGA
	U	GGAGGATAGTTGGATTGAGTTAATGTT	CAAATCCCTCCCAAACACCAA
*APAF1*		CCCAGAGGCTTCCACTTAATATTG	CAAACATCATCCAAGATCAAGAGAGA
*BAX*		TGAGTACTTCACCAAGATTGCCA	AGTCAGGCCATGCTGGTAGAC
*BCL2L11*		TGTCTGACTCTCTCCGGACTG	TGACCACATCGAGCTTTAGCCAGTCA
*BNIP3*		CCAGAACATCATCCCTGCAT	TTCCAGTAGGGTCTCGACTTG
*BNIP3L*		CTCAGGATCCACAGCAAACA	CCAGACTGGACTCTGCCTTC
*DNMT1*		*CCATCAGGCATTCTACCA*	CGTTCTCCTTGTCTTCTCT
*DNMT3a*		TATTCATGAGCGCACAAGAGAGC	GGGTGTTCCAGGGTAACATTGAG
*STAT3*		CACCTTGGATTGAGAGTCAAGAC	AGGAATCGGCTATATTGCTGGT

*M* methylated, *U* unmethylated

#### Bisulfite sequencing

Bisulfite sequence assay was performed to demonstrate the methylation status of DAPK1 promoters in the cell line. Genomic DNA from cell lines was isolated with DNeasy kit (Qiagen, Valencia, CA, USA). Genomic DNA was subjected to bisulfite conversion with Epitect Bisulfite kit (Qiagen, Valencia, CA, USA) according to the manufacturer’s instructions. The BSP primers for DAPK1 promoter region were 5′- GAGGTTTTTAGTGGATATGGGATT-3′ (sense) and 5′-TCCACCTCCAAAATTCAAATAATT-3′ (antisense), designed by Methyl Primer Express v1.0. Amplified PCR products were purified and sequenced on an ABI Prism 3500 Genetic Analyzer.

### Extraction and expression of acetylated histone proteins

Leukemia cells HL60 and U937 were treated with 50 μmol/L Qu for 48 h and were harvested and washed twice with ice-cold phosphate-buffered saline (PBS) supplemented with 5 mM sodium butyrate. After washing, cells were resuspended in Triton extraction buffer [PBS containing 0.5% Triton X-100 (vol/vol), 2 mM phenylmethylsulfonyl fluoride, 0.02%(wt/vol) NAN3] and lysed on ice for 10 min with gentle stirring, centrifuged at 2000 r.p.m. for 10 min at 4 °C. The pellet was washed in Triton extraction buffer and then resuspended in 0.2 N HCl. Histones were acid extracted overnight at 4 °C and centrifuged at 2000 r.p.m. for 10 min at 4 °C. Samples were processed for the analysis of histones using immunoblotting.

### Western blot analysis

Total cell protein was extracted in RIPA buffer. Protein concentrations were quantified by the Bio-Rad Protein Assay Kit. Equal protein amounts were loaded on 8 to 15% SDS polyacrylamide gels and electrophoretically transferred to nitrocellulose membrane. Nonspecific binding sites were blocked by incubation with a buffer containing Tris (10 mmol/L, pH 7.4), NaCl (150 mmol/L), Tween 20 (0.1%), and fat-free dry milk (5%). Membranes were incubated overnight with a specific primary antibody, at 4 °C, followed by horseradish peroxidase-conjugated secondary antibody, at room temperature for 1 h. Immunoreactivities were visualized by ECL Western Blot Analysis System (Amersham Pharmacia Biotech).

### Posttranscriptional histone modifications

#### Chromatin immunoprecipitation (ChIP)

*Apoptosis ChIP human PCR array* (SAbiosciences, Qiagen) was used to evaluate whether Qu treatment induces an enrichment of modified histones such as acetylated histone 3 and histone 4 (H3ac, H4ac) in the promoter regions of genes associated to apoptosis pathway. After cross-linking cells with formaldehyde, chromatin containing covalent complexes of genomic DNA and nuclear factors were isolated and sheared by sonication into fragments of between 500 and 1500 bp. The DNA sequences and the posttranslational histone modifications were then immunoprecipitated with specifically antibodies for anti H3ac and H4ac. Next, cross-linking was reversed followed by nucleic acid purification to detect DNA by real-time PCR.

### RNA extraction and real-time PCR

The cells treated with Qu and the samples from the xenotransplants were collected, snap frozen, and stored at − 80 °C in RNAlater® (Qiagen, Valencia, CA, USA). Total RNA was isolated using the miRNeasy mini kit® (Qiagen). Single-stranded cDNA was synthesized from the RNA using the high capacity cDNA archive kit® (Applied Biosystems, Foster City, CA, USA) following the manufacturer’s protocol.

Quantitative PCR (Sybr Green®) was performed on a 7500 real-time PCR system (Applied Biosystems) using threshold cycle numbers as determined by the RQ Study software (Applied Biosystems). The reactions were run in triplicate, and the threshold cycle numbers were averaged.

*DNMT1*, *DNMT3a*, *STAT3*, *DAPK1*, *BCL2L11*, *BAX*, *BNIP3*, *BNIP3L*, and *APAF1* expressions were measured and normalized using *HPRT* as an endogenous control. The relative expression was calculated according to the formula 2^(−∆∆Ct)^ [40], and the results were expressed as average gene expression ± SD.

### Human tumor xenograft model

Female (NOD.CB17-Prkdc^scid^/J lineage) 8–11-week-old animals, from the Jackson Laboratory, bred at the Animal Facility Centre at The University of Campinas, under specific pathogen-free conditions, were matched for bodyweight before use. Animal experiments were performed following institutional protocols and guidelines of the Institutional Animal Care and Use Committee. Mice were inoculated, s.c, in the dorsal region, on day 0 with 0.1 mL of HL60 or U937 cell suspension (1 × 10^7^ cells/mice). Every 7 days, tumor volumes were evaluated according to the formula: tumor volume (mm^3^) = (length × width^2^)/2. Qu treatment (HL60 xenograft model, treated group *n* = 5; U937 xenograft model, treated group *n* = 7) was initiated after tumors reached 100 to 200 mm^3^ and was administered once every 4 days by i.p injection at 120 mg/kg body weight. The control group (HL60 xenograft model *n* = 4; U937 xenograft model *n* = 5) received equal amounts of vehicle solution, as previously described [41]. Mice were sacrificed after 21 days. Tumors were then removed, minced, snap frozen, and stored at − 80 °C in RNAlater® (Qiagen, Valencia, CA, USA).

### Statistical analysis

The results of the real-time PCR were expressed as means ± SD. All the experiments were realized in triplicate. Student’s *t* test was used to determine the statistical difference between treated and control group. The data were considered significant if *p* < 0.05.

## Abbreviations

AML: Acute myelogenous leukemia
BCL2L11: BCL2-interacting mediator, (*BIM*)
CML: Chronic myeloid leukemia
ChIP: Chromatin immunoprecipitation
DAPK1: Death-associated protein kinase 1
DNMTs: DNA methyltransferases
DNMT1: DNA methyltransferase 1
DNMT3a: DNA methyltransferase 3a
HATs: Histone acetyltransferases
H3ac: Acetylated histone 3
H4ac: Acetylated histone 4
HDACs: Histone deacetylases
HDACi: Histone deacetylase inhibitors
IM: Imatinib
MDSs: Myelodysplastic syndromes
MSP-PCR: Methylation-specific PCR
ncRNA: Noncoding RNAs
Qu: Quercetin
SAHA: Suberoylanilide hydroxamic acid
